# A Detailed Analysis of the Murine TAP Transporter Substrate Specificity

**DOI:** 10.1371/journal.pone.0002402

**Published:** 2008-06-11

**Authors:** Anne Burgevin, Loredana Saveanu, Yohan Kim, Émilie Barilleau, Maya Kotturi, Alessandro Sette, Peter van Endert, Bjoern Peters

**Affiliations:** 1 Institut National de la Santé et de la Recherche Médicale, Paris, France; 2 Université Paris Descartes, Faculté de Médecine René Descartes, Paris, France; 3 La Jolla Institute for Allergy and Immunology, La Jolla, California, United States of America; Karolinska Institutet, Sweden

## Abstract

**Background:**

The transporter associated with antigen processing (TAP) supplies cytosolic peptides into the endoplasmic reticulum for binding to major histocompatibility complex (MHC) class I molecules. Its specificity therefore influences the repertoire of peptides presented by MHC molecules. Compared to human TAP, murine TAP's binding specificity has not been characterized as well, even though murine systems are widely used for basic studies of antigen processing and presentation.

**Methodology/Principal Findings:**

We performed a detailed experimental analysis of murine TAP binding specificity by measuring the binding affinities of 323 peptides. Based on this experimental data, a computational model of murine TAP specificity was constructed. The model was compared to previously generated data on human and murine TAP specificities. In addition, the murine TAP specificities for known epitopes and random peptides were predicted and compared to assess the impact of murine TAP selectivity on epitope selection.

**Conclusions/Significance:**

Comparisons to a previously constructed model of human TAP specificity confirms the well-established differences for peptide substrates with positively charged C-termini. In addition these comparisons show that several residues at the N-terminus of peptides which strongly influence binding to human TAP showed little effect on binding to murine TAP, and that the overall influence of the aminoterminal residues on peptide affinity for murine TAP is much lower than for the human transporter. Murine TAP also partly prefers different hydrophobic amino acids than human TAP in the carboxyterminal position. These species-dependent differences in specificity determined *in vitro* are shown to correlate with the epitope repertoire recognized *in vivo*. The quantitative model of binding specificity of murine TAP developed herein should be useful for interpreting epitope mapping and immunogenicity data obtained in humanized mouse models.

## Introduction

CD8^+^ T lymphocytes monitor cells for non-self protein expression by scanning their surface for MHC class I molecules presenting peptides derived from proteins expressed inside the cells [1]. The recognition of peptide epitopes by T cells mediates the clearance of viral infections [2]–[4], controls the growth of intracellular bacteria [5]–[8], and has a role in immunity against some types of cancer [9]. Defects in self tolerance can lead to CD8^+^ immune responses that contribute to autoimmune pathologies [10]. The importance of CD8^+^ immune responses for human health has motivated many *in vivo* studies aiming to identify the peptide targets of CD8^+^ responses. As studies with human patients are often not ethically feasible and samples can be hard to obtain, many epitope discovery studies have been conducted in humanized mice [11]–[13]. It is therefore important to understand differences between murine and human antigen processing machinery that may affect the identity and immunodominance of HLA class I-restricted peptide epitopes.

The majority of peptides recognized by CD8^+^ T cells are generated through the endogenous MHC-I antigen processing and presentation pathway. Initially proteins in the cytosol are cleaved into peptide fragments by proteasomes, possibly in concert with TPPII [14], [15], and by other proteases. The produced peptides are subject to rapid degradation by cytosolic aminopeptidases, and only approximately 1% of the peptides [16], [17] escape degradation through transport into the ER by the TAP transporters that prefer peptides with a length of 8 to 16 residues [18]–[20]. Inside the ER, peptides are subject to further N-terminal trimming by ERAP1, which efficiently cleaves substrates between 8 and 16 residues in length [21]. In humans, an additional ER aminopeptidase, ERAP2, with a preference for basic residues, complements ERAP1 [22]. Finally, peptides with suitable length and sequence are able to bind empty MHC class I molecules with the help of multiple chaperones forming the MHC class I loading complex. The peptide:MHC complex is then transported to the cell surface through the Golgi apparatus. Sequence specificities at each step in this antigen processing pathway influence what peptides are eventually presented to T cells.

The focus of the present study is the murine TAP transporter, a heterodimeric complex consisting of the TAP1 and TAP2 proteins, both of which are members of the ATP binding cassette (ABC) transporter family [23]. Peptide transport by TAP is a sequential process initiated by peptide binding to a site probably located at the interface between the cytosol and the transmembrane channel of TAP, followed by ATP dependent transport of the peptide into the ER [24]. Two assays measuring peptide affinity for TAP are available. One of these measures the ATP- and temperature-dependent accumulation of glycosylated transported peptides in the ER [25]. This assay has the advantage of measuring the complete peptide transport process, but may also be affected by the rate of peptide degradation in the cytosol either before transport into the ER, or after retrograde transport out of the ER [18], [26], [27]. While cytosolic peptide degradation is generally extremely rapid, some peptides, for example those with multiple basic residues in the aminoterminal positions, have been found to be more resistant to degradation [15], [28]. A second assay measures only the initial peptide binding step at low temperature, rendering interference by peptidases less likely [20]. While it was theoretically conceivable that some peptides bind TAP but are not transported, which would have rendered the latter assay unreliable, it has been found that addition of very long side chains is required to produce peptides that bind TAP without being transported [29]. Moreover, it has been directly demonstrated that peptide binding affinity reflects peptide transport affinity [30], [31]. Further strong evidence for the biological relevance of the results of TAP binding assays was provided in a study showing that TAP binding affinity paralleled closely the efficiency of epitope presentation by cell surface class I molecules [17]. The fact that an algorithm that is based on the TAP affinity of a large number of peptides measured using the binding assay, ameliorates prediction of naturally processed CTL epitopes represents an additional corroboration of the binding assay [32]

The first *in vitro* studies characterizing the sequence specificity of TAP peptide transport focused mainly on C-terminal peptide residues. Murine and rat cim^b^ TAP molecules prefer peptides with hydrophobic C-termini [19], [33], [34], while human TAP molecules transport efficiently peptides with hydrophobic and basic C-terminal residues [34]. Additional studies of the influence of the aminoterminal and internal peptide sequence, carried out using the peptide transport assay, concluded that these positions have little effect on TAP affinity, with the exception of Pro residues in several positions which decreased transport efficiency [34]–[36]. However, detailed studies of human TAP specificity using the peptide-binding assay elucidated a significant influence of the three N-terminal peptide residues [31], [32], [37], [38]. The same preferences are found in peptides of different lengths for the first three N-terminal and the C-terminal positions. This makes it likely that peptide binding to TAP involves two contact sites at the free peptide ends, with each having a defined binding preference, while the connecting residues can form loops of variable lengths [37]. This model would also explain why there is a minimal peptide length required for efficient transport [18]–[20].

In summary, while past studies with model substrates have demonstrated differences between murine and human TAP specificity, subsequent large-scale studies that allow for the development of general quantitative prediction models have focused on human TAP alone. In this study, we have therefore characterized the binding specificity of murine TAP with a large panel of peptides, allowing for a detailed comparison between the transport preferences in the two species. We have further utilized this data to build a predictive model of TAP transport for peptides of any length, which can be applied to identify likely epitopes or their precursors.

## Results

### Establishing the murine TAP binding assay

**Figure 1:**
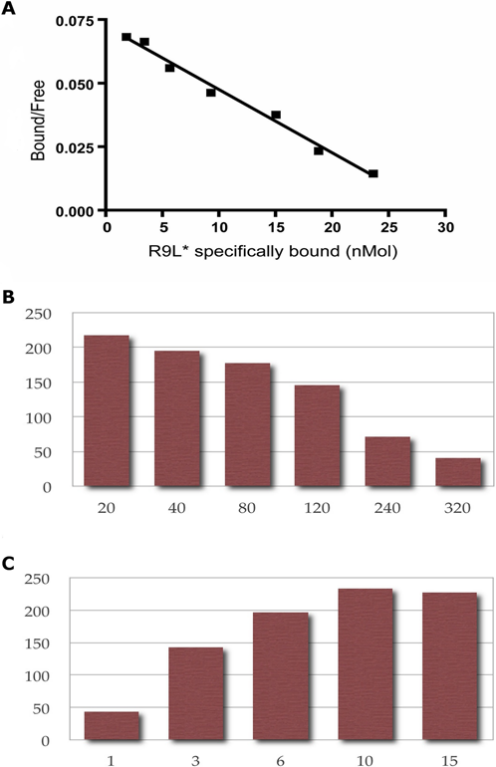
Establishing the murine TAP binding assay. Panel A depicts a Scatchard plot analysis of ^125^I-labeled peptide binding to murine TAP1/TAP2 complexes. The lower panels depict dilution series determining the optimal concentrations for (B) the fluorescent reporter peptide and (C) the volume of microsomes expressing murine TAP. Maximal specific binding, as measured by the polarization reading (y-axis), is achieved at 20 nM reporter peptide and 10 µl of microsomes.

We previously established an assay measuring competition for binding to human TAP complexes, over-expressed in insect cells, between a radio-labeled reporter peptide and unlabeled test peptides [20]. In this study, standard procedures were utilized to produce recombinant baculoviruses driving expression of the murine TAP1 and TAP2 transporter subunits, as confirmed by immunoblot analysis (not shown). Formation of TAP1/TAP2 complexes able to bind peptides was measured using peptide R9L (RRYNASTEL) as a standard reporter [20], labeled with ^125^I. **Figure 1** shows a Scatchard plot analysis of ^125^I-R9L binding to microsomes from Sf9 cells co-infected with mouse TAP1 and TAP2 viruses. This experiment indicates 8×10^6^ binding-competent TAP complexes per Sf9 cell, and a K_D_ of 400 nM for the reporter peptide. Previously established values for human TAP complexes expressed in Sf9 cells are 1×10^6^ and 390 nM, respectively [20]. Thus, mouse TAP complexes are expressed with very high efficiency in our insect cell system, and bind the standard reporter peptide with the same affinity as human TAP complexes.

Using the same insect cell system for TAP expression, a novel assay was established which is based on detecting fluorescence polarization rather than the activity of a radioactive tracer. A reporter peptide was synthesized in which position 6 (Ser) of peptide R9L was substituted by a Cys residue coupled to a fluorescein group (R9L-FITC). As expected [20], this peptide did not bind to microsomes containing either murine TAP1 or TAP2 protein alone (data not shown). To establish a fluorescence polarization assay, we determined the concentrations of reporter peptide and microsomes yielding the highest specific binding signal (mP reading). Optimal results were obtained with a peptide concentration of 20 nM and a microsome volume of 10 µl (**Figure 1**).

Table 1 shows an example of assay results, with five peptides at each end of the range of affinities measured in the assay. Measured relative affinities range over more than five logs. As expected from previous characterizations of murine TAP specificity, many peptides with very low affinities have a C-terminal Lys residue, while high affinity peptides have a hydrophobic C-terminus.

**Table 1:** Highest and lowest measured murine TAP affinities

Sequence	relative IC_50_
AAFEFINSL	0.11
RDAEFVMCL	0.15
GTHVLLPFY	0.16
NLYISDYKM	0.23
AIITPVVFY	0.25
LLAVCGCIE	>5000
NIVYKKNNR	>5500
KTGGPIYKR	>5800
NMEANDPEK	>6000
NQSSHKGVG	>7000

Thus, the combination of very high-level expression of mouse TAP complexes in Sf9 microsomes, and high affinity binding of the reporter peptide R9L-FITC to these complexes, allowed us to establish a highly efficient novel binding assay for characterizing the specificity of mouse TAP transporters. Of note, this assay not only avoids the use of radioactive tracers but also is significantly faster than our previous assay since microsome washing for removal of free peptide ligand is not required.

The fluorescence polarization assay was validated by comparing data obtained using insect cell-expressed human TAP complexes and reporter peptide R9L-FITC (which binds to human TAP with identical affinity), with data previously generated in the radioligand binding assay. These experiments showed that peptide affinities determined in the two assays were within the margin of experimental error of each other (data not shown).

### Selecting and testing peptides for binding to murine TAP

We selected and tested peptides for binding to murine TAP binding in a two-stage process. In the first stage, we selected a set of 198 peptides 9 residues in length, which included several known murine T cell epitopes and other peptides chosen from natural protein sequences. These peptides were chosen so to ensure that all amino acids were represented at all nine positions in at least two peptides of the dataset.

In the second stage, we selected a set of peptides using a “query by committee” (QBC) approach [39], [40], which should minimize any holes in knowledge left after the first set. Briefly, five prediction models were generated, each based on randomly drawn 80% of the current binding data. These five models were used to predict the binding affinity of 50,000 peptides of length nine. Thirty-three peptides were chosen that had an at least 25-fold difference between the highest and lowest of the five predicted IC_50_ values. Such peptides represent likely holes in knowledge, because no robust prediction for their IC_50_ value was determined from the initial set of peptides. In total, we determined the binding affinities of 231 peptides of length 9, which are listed in Table S1.

### Establishing a predictive model for 9-mer binding to murine TAP

**Figure 2:**
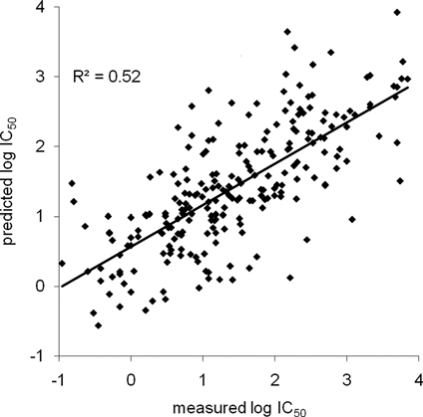
Assessing prediction quality through cross-validation. Each point in the scatterplot represents one peptide. For each peptide, the measured affinity (x-axis) is plotted against the predicted affinity (y-axis). The predicted values were obtained in a blinded 5-fold cross-validation.

We utilized the set of measured binding affinities to generate a prediction model of murine TAP specificity, and evaluated its accuracy. The SMM-BP algorithm was used to determine a scoring matrix of murine TAP specificity. Briefly, the algorithm determines the matrix by minimizing the difference between measured and predicted affinities, while preferring matrices that assign similar scores to amino acids known to have similar binding characteristics (see Material & Methods). To evaluate the quality of the binding prediction, we used 5-fold cross validation: the peptides were randomly partitioned into 5 mutually exclusive subsets. For the validation, one of the 5 subsets was used as the test set and the other 4 subsets were combined to form a training set. A prediction was generated using only peptides from the training set, and used to predict the affinity of all peptides in the blind set. Repeating this process 5 times gives one affinity prediction for each peptide in the original set, which can be compared to its known measured affinity. **Figure 2** depicts the results of the cross validation. The linear correlation between measured and predicted affinities was highly significant (Pearson r^2^ = 0.52, p<0.0001). 85% of the peptides had a measured affinity within 10-fold range of the predicted IC_50_ value. In conclusion, we have established a matrix model of 9-mer peptide binding to murine TAP that correlates well with measured affinities in a blinded evaluation.

**Figure 3:**
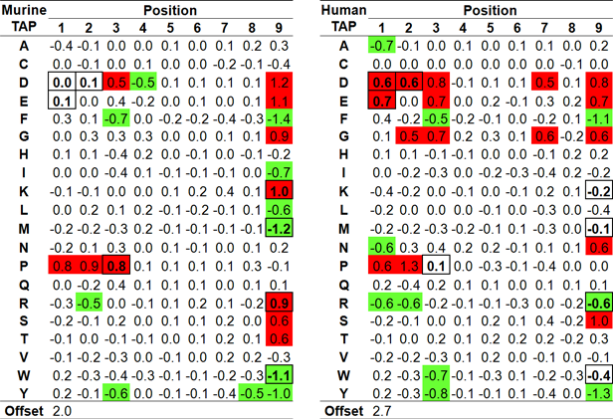
Binding specificity of murine and human TAP. The matrices were generated from peptide binding data to murine (left) or human (right) TAP molecules. Each column in the matrix corresponds to a position in a peptide of length 9, and each row to a certain residue. Negative values (marked in green for x<−0.5) correspond to residues with higher affinities, while positive values (marked in red for x>0.5) correspond to lower affinities. Matrix positions that differ by more than 0.55 between the two species are boxed.

Next, we examined the influence of specific peptide residues on binding to murine TAP determined in our matrix model. Figure 3a shows the prediction matrix for murine TAP that assigns a score to each residue in a 9-mer peptide. The scores approximate the contribution to binding free energy of the corresponding peptide residue. Residues at peptide positions 1, 2, 3 and 9 (the C-terminus) have the greatest influence on binding to murine TAP, similar to previous observations for human TAP [31]. At the C-terminus, hydrophobic residues are the most preferred for murine TAP, in agreement with previous reports [19], [34]. At positions 1,2 and 3, the most notable impact on binding is the strong negative influence of proline which had also been reported before [34], [36]. The remaining matrix entries with significant scores (R2, D3, F3, Y3, D4 and Y8) were not singled out as influential residues in the one study looking at the impact of those positions [36]. This is likely due to their lower impact compared to the C-terminal and Proline residues, as well as the fact that several residues including D and R were missing from the panel tested in [36]. Overall, the specificity matrix calculated by us for murine TAP is in good qualitative agreement with previously published experimental data generated with model substrates.

In order to identify residue positions contributing to the difference between murine and human TAP specificity, we compared the murine matrix scores with those of a previously determined human TAP specificity matrix [32]. Figure 3 depicts the two matrices side by side, and identifies residues with absolute score differences above 0.55 with a bold border. The most drastic difference between matrix scores was observed for Lysine (K) and Arginine (R) at the C-terminus. This corresponds to the previously described finding that only human but not murine TAP complexes can bind peptides with positively charged C-termini [34], [41]. Methionine (M) and Tryptophane (W) are strongly preferred residues for binding at the C-terminus of murine TAP, while they have little or no effect for human TAP. Moreover, compared to the human TAP matrix, the charged residues D and E are essentially neutral in positions 1 and 2 of murine TAP, whereas they clearly inhibit binding to human TAP. Finally, Proline (P) at position 3 disrupts binding to murine TAP while being essential neutral for the human transporter. In summary, although the relative weight of the aminoterminal positions differs between human and murine TAP (see below), binding affinity for both depends mainly on the three N-terminal and the C-terminal residues of a peptide. While the two transporters share many preferences, there are a number of residues with clearly distinct binding patterns.

Next we examined the overall importance of C-terminal and N-terminal peptide residues for binding to human and mouse TAP. Mouse TAP has strong preferences or aversion for 13 of 20 amino acids at the C-terminus, while human has only 7. Conversely, human TAP shows preferences or aversion for 16 amino acids in positions 1 to 3 (6 in P1, 4 in P2, 6 in P3), while mouse TAP displays only 7 (1 in P1, 2 in P2, 4 in P3). At the C-terminus, this difference is statistically significant when comparing the absolute coefficient values (p = 0.030, Mann-Whitney Test). For the three N-terminal residues, the p value is 0.062, just slightly above the customary cutoff of p = 0.05.

To quantify the relative impact of N-terminal residues and the C-terminus with a single number, we calculated the ratio of standard deviations for the scores for human and mouse TAP at the corresponding positions. For human TAP, the standard deviation of the C terminal scores is 0.59 and 0.41 at the N-terminus, which gives a ratio of 1.4. In contrast, for murine TAP the standard deviation at the C-terminus is 0.82 and 0.31 at the N-terminus, corresponding to a ratio of 2.6 which is nearly twice as high. Overall this shows that murine TAP selects its ligands much more on the basis of the C terminus than its human counterpart.

### Predicting peptide binding for varying lengths

**Figure 4:**
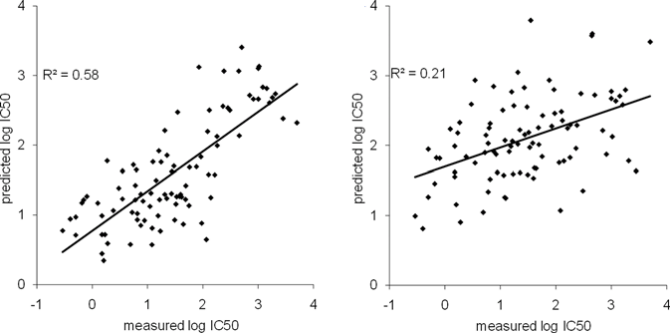
Prediction quality for peptides of varying lengths. Each scatterplot represents predicted vs. measured affinities for the set of 8, 10 and 11-mer peptides tested for binding to murine TAP. The predictions on the left plot were made with the murine TAP prediction matrix, the one in the right plot using the previously established human TAP matrix.

As TAP is known to transport precursor peptides longer than nine residues into the ER, we examined if we could predict their affinity using an approach we had previously developed for human TAP. This was based on representing peptides of any length by their three N-terminal residues plus their one C-terminal residue, and assigning them a binding score based on matrix columns 1, 2, 3 and 9 of the matrix for 9-mer peptides. To evaluate this approach, we first tested a set of 92 peptides with length 8, 10 and 11 for binding to murine TAP (Table S1 **).** **Figure 4** shows scatterplots of the predicted versus measured affinities for each of these peptides, using either the human or the murine matrices as predictors. The correlation between prediction and measurement is highly significant for the murine matrix predictions (r^2^ = 0.58, p<0.01), even slightly above the correlation determined above in cross-validation using 9-mers alone. The results for individual peptide sets are similar, with 8-mers, 10-mers and 11-mers having correlations coefficients r^2^ of 0.53, 0.67 and 0.64, respectively. In addition, we have compared how the human TAP matrix would have performed on this peptide set. As shown in **Figure 4** **,** predictions based on human TAP data would have correlated very poorly with the experimental results (r^2^ = 0.21). Overall, this confirms that our approach can be used to predict peptide binding for multiple lengths to murine TAP, and that murine TAP has a distinct binding pattern from that of human TAP.

### Prediction of T-cell recognition

**Figure 5:**
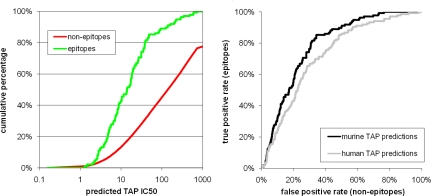
Utilizing TAP transport predictions to identify T cell epitopes. Peptides from LCMV and VACV were classified into either epitopes or non-epitopes depending on their recognition by T cells in mice post infection (see Methods). In the left panel, murine TAP affinity predictions were made for all peptides. At varying cutoffs, the cumulative percentage of epitope and non-epitope peptides that have an affinity of at least the cutoff is given. In the right panel, an ROC plot is used to directly compare the murine and human predictions. The curve made with murine TAP predictions is consistently better at separating murine epitopes from non-epitopes than the human one.

Next, we tested if our murine TAP transport predictions could identify which peptides contained in a pathogen are recognized as T cell epitopes. As a test set, we used epitopes identified in H-2^b^ or H-2^d^ mice following viral infections with LCM or Vaccinia virus (described in [42], [43] and Oseroff, manuscript submitted). As T cell priming during infection requires processing and presentation of peptides in infected target cells, peptides that are recognized post infection are presumed to be at least permissible for TAP transport. A total of 139 epitopes restricted by H-2 D^b^, K^b^, D^d^, K^d^ and L^d^ were included in this set, including 34 8-mers, 96 9-mers, 6 10-mers and 3 11-mers. For each virus, we predicted the TAP affinity of all 8, 9, 10 and 11mer peptides that can be derived from their viral source proteins. In Figure 5, these predicted affinities are compared for peptides recognized as T cell epitopes and non-epitopes. As expected, T cell epitopes showed markedly higher TAP affinities than non-epitopes. For example, 43% of all epitopes have a predicted IC_50_ <10 nM, while only 14% of non-epitope peptides have a similar high affinity. Overall, this confirms that a peptide with high predicted murine TAP affinity has an increased probability of being recognized by T cells following viral infections in mice.

We tested if the differences we identified between human and murine TAP specificity influences the epitope repertoire recognized by human and murine T cells. If this is indeed the case, the correlation between predicted murine TAP affinity and T cell recognition in mice should be higher than for predicted human TAP affinity. We utilized ROC curves to directly compare the ability of both predictions to identify murine T cell epitopes (Figure 5), which clearly demonstrated superior performance for the murine TAP prediction. This confirms that the murine TAP predictions are superior to human ones at identifying epitopes recognized in mice.

For human TAP, we had previously shown that taking the transport of epitope precursors into account can greatly improve the prediction quality [32]. We applied the same approach to murine TAP by averaging the score of precursor peptides up to a maximal extension N_max_, and assigning a weighting factor α. We tested the performance of these predictions on the same dataset used in Figure 5 for varying values of N_max_ and α. The prediction performance was only marginally improved, below the threshold for statistical significance for all parameter combinations. Thus, the previously established approach to improve epitope predictions for human TAP by including the transport of precursors was not successful when applied to murine TAP and the prediction of viral epitopes. This demonstrates that the predominant influence of C-terminal peptide residues on murine TAP affinity reduces the impact of different N-terminally prolonged precursors being transported on epitope recognition in mice.

## Discussion

We here presented a large-scale analysis of murine TAP specificity obtained using TAP binding assays. The quantitative binding affinity of 231 9-mer peptides was determined, and used to computationally derive murine TAP specific scoring matrices. The resulting specificity pattern is in good agreement with previous publications that used peptide substitution libraries to directly compare different residues in one position on the affinity or transport rates of murine TAP[19], [34], [36]. In addition, the present study provides the first quantitative specificity data for over seventy residue/position combinations not covered by published substation library data. This complete coverage ensures that no residue/position combination that strongly influences binding has been overlooked, which is necessary to quantitatively predict murine TAP binding specificity for any peptide sequence.

We took advantage of being able to compare human and murine TAP specificity matrices, and found that several residues at the N-terminus of peptides that strongly influence binding to human TAP showed little effect on binding to murine TAP. This includes a complete lack of any residue with a strong positive effect on binding in position 1. In contrast, for peptide C-termini, murine TAP is more specific in its binding preference than human TAP. Taken together, we showed that murine TAP is more skewed than human TAP towards binding peptides based on their C-terminus alone. While not reported as significant in the original publications, examining the figures in references [34], [36] supports such a conclusion.

The differences discovered between human and murine TAP binding specificity were shown to correlate with differences in the ability to predict epitope recognition in murine hosts. This demonstrates that our *in vitro* studies correlate with antigen processing events *in vivo*. It also reinforces that studies of epitope repertoire in mice and human need to take differences between their TAP transporters into account.

As TAP is known to transport epitope precursors up to a length of about 16 residues, it is important to characterize its substrate specificity for varying lengths. We were able to successfully predict the affinity of peptides between 8 and 11 residues in length by modeling their binding interaction at the C terminus and the three N terminal residues. In this model of binding, the connecting residues 4 to C-1 of longer peptides are assumed to make only weak interactions with the TAP molecules. This model was previously applied to human TAP, and is shown for the first time to apply to murine TAP as well.

The description of murine TAP specificity provides one crucial component towards explaining species specific differences in epitope recognition, which could explain differences in epitope repertoire in humans and HLA transgenic mice frequently used in epitope discovery and vaccine development studies. These data likely will have to be complemented by other studies directed at understanding the impact of other components of the antigen processing pathway that are known to differ between the two species. This includes the lack of ERAP2 in mice, and incompatibilities between human MHC molecules and the murine peptide loading complex (specifically tapasin [44]). Also, the difference in mouse and human self, which, due to the requirement for self-tolerance, leads to differences in TCR repertoire, may have to be taken into account. Finally, genomic and proteomic tools will permit to study host influences on viral protein expression, or host specific viral immune evasion mechanisms [45], which will also affect the epitope repertoire in a species specific manner.

In conclusion, our detailed analysis of the binding specificity of the murine transporters allows for a sensitive comparison between peptide selection by mouse and human TAP, and demonstrates that epitopes recognized by murine CTLs are selected for increased TAP affinity. This work provides a key step towards the complete and differential description of human and murine antigen processing events.

## Materials and Methods

### Preparation of insect cell microsomes over-expressing murine TAP complexes

Microsomes were prepared precisely as described previously for microsomes expressing human TAP. Briefly, full-length cDNAs encoding the murine TAP transporters were amplified from plasmids pFM370.1 and pFM372.3 (kindly provided by Frank Momburg), sequenced, and inserted as XbaI/NotI or BamhI/XbaI fragments, respectively, into the baculovirus transfer plasmid pVL1393, and recombinant viruses were produced by co-transfection of BaculoGold™ (Pharmingen) linearized baculovirus DNA together with the plasmids into Sf9 cells, followed by plaque assays and isolation of clones containing TAP DNA. For production of microsomes, 175 cm^2^ cell culture flasks each containing 5–8×10^6^ Sf9 cells were infected at a multiplicity of infection of 3 with high titer viral supernatant. Three days later, cells were harvested, broken up in a Douncer, and fractionated using a sucrose step gradient, as described [20]. One flask of infected cells yielded on average 1 ml of microsome solution.

### Binding Assay

The Scatchard plot shown in Fig. 1 was carried out with ^125^I-labeled reporter peptide RRYNASTEL, using exactly the same conditions as in the Scatchard plot analysis shown in [20]. The fluorescence polarization assay was carried out entirely at 4°C according to the following protocol. Peptides were obtained at ∼80% purity (Pepscan or Sigma-Genosys); peptide purity was controlled by HPLC, and identity verified by mass spectrometry. Peptide stocks were prepared at 10 mM in pure DMSO (50% DMSO in H_2_O for peptides with cysteines). Serial dilutions of test peptides were prepared in assay buffer (PBS with 1 mM dithiotreitol, 2 mM MgCl_2_ and 0.1% BSA), such that molar excesses relative to reporter peptide between 0.1× and 1000× could be tested. Dilutions of test peptides and reporter peptide (final concentration 20 nM) were added to black flat bottom 96-well plates (FluoroNunc™, Nunc) in a total volume of 150 µl. Then 10 µl of microsomes, diluted to 50 µl in assay, buffer, was added, and fluorescence polarization immediately read using a Mithras LB940 microplate reader (Berthold, Thoiry, France). Unlabeled reporter peptide R9L was included in each assay as competitor to allow for normalization of data between different assays. Specific binding was determined as the difference between the polarization (mP) reading in the absence of competitor to the reading in the presence of a 1000-fold excess of unlabeled reporter peptide. The molar excess of the test peptide required for inhibiting 50% of specific binding of the reporter peptide was then determined (IC_50_). Assay results were finally expressed as ratio, i.e. IC_50_ of the test peptide divided by the IC_50_ of the unlabeled reporter peptide, in order to normalize results among different assays. Each peptide was tested in at least two independent assays; if the two results diverged by more than 25 percent, additional assays were performed. The final result corresponds to the mean of all validated assay results.

### Removal of Outliers

A total of five peptides with the sequences ILMIFISSFL, AFILGIIITV, LMYIFAAL, MIVAWFLLL and WAIINTIYF were removed from the dataset. For each of these peptides, relatively weak or no competitive binding to murine TAP could be determined, even though their sequence strongly suggests them to be at least reasonable binders. At the same time, these peptides are very hydrophobic and therefore likely to have problems with solubility. This would result in them being classified as poor binders in the assay we employed, which is the basis for their removal.

### SMMBP Method

Peptide binding predictions for TAP and MHC-I molecules were carried out using the method SMM-BP (manuscript in preparation), an improved version of SMM [32], [46] that includes a Bayesian Prior (BP). Briefly, this method adds prior knowledge of amino acid similarities to the matrix determination process, by including a general amino acid binding covariance matrix. This covariance matrix was determined directly from a set of 180 randomized peptide libraries of length nine with measured binding affinities of to 24 MHC-I alleles from human, chimpanzee, macaque, and mouse (compare [47]). The SMM-BP method optimizes a scoring matrix such that the squared differences between measured and predicted binding affinity values are minimized, which corresponds to maximizing the likelihood of observing these measurements giving the prediction matrix and assuming a normal distribution of differences between predicted and measured values. The prior probability of the scoring matrix is calculated directly from the amino acid covariance matrix, which assumes that the scoring matrix follows the same Multivariate normal distribution. The final matrix is calculated following Bayes' Theorem by maximizing the product of the probabilities of likelihood and prior.

### T cell epitope dataset

The epitope dataset used were taken from ([42], [43] and Oseroff et al, manuscript submitted). The epitope mapping in the three studies was performed as follows: C57BL/6 or Balb/c mice were infected with LCMV Armstrong or VACV WR. Eight to ten days post infection, mice were sacrificed, and splenocytes were taken. The cells were tested directly ex vivo in a murine IFN-γ ELISPOT assays for responses against peptide pulsed target cells. The tested peptides were selected by their predicted ability to bind the H-2 D^b^, D^d^, K^b^, K^d^ or L^d^ molecules. Responses were measured in triplicate, and experiments repeated at least twice. Peptides that consistently gave responses twice above background and with SFC/10^6^>20 were considered positive. The mice purchased from The Jackson Laboratory and maintained at the La Jolla Institute for Allergy and Immunology facility (San Diego, CA) following National Institutes of Health guidelines and Institutional Animal Care and Use Committee approved animal protocols.

## Supporting Information

Table S1 Experimentally Measured Murine TAP Binding Affinities for the Peptides.
(0.04 MB XLS)
